# Childhood Sexual Violence and Consistent, Effective Contraception Use among Young, Sexually Active Urban Women

**DOI:** 10.3390/bs5020230

**Published:** 2015-05-22

**Authors:** Deborah B. Nelson, Stephen J. Lepore, Dimitrios S. Mastrogiannis

**Affiliations:** 1 Temple University College of Public Health 1301 Cecil B. Moore Avenue Ritter Annex Room 905, Philadelphia, PA 19122, USA; 2 Temple University College of Public Health 1301 Cecil B. Moore Avenue Ritter Annex Room 957, Philadelphia, PA 19122, USA; 3 Department of Obstetrics and Gynecology, University of Illinois at Chicago, 820 South Wood Street, M/C 808 Chicago, IL 60612, USA

**Keywords:** contraception use, childhood sexual violence, depressive symptoms, self-esteem, unintended pregnancy

## Abstract

Unintended pregnancy (UP) is a significant public health problem. The consistent use of effective contraception is the primary method to prevent UP. We examined the role of childhood sexual and physical violence and current interpersonal violence on the risk of unintended pregnancy among young, urban, sexually active women. In particular, we were interested in examining the role of childhood violence and interpersonal violence while recognizing the psychological correlates of experiencing violence (*i.e.*, high depressive symptoms and low self-esteem) and consistent use of contraception. For this assessment, 315 sexually active women living in Philadelphia PA were recruited from family planning clinics in 2013. A self-administered, computer-assisted interview was used to collect data on method of contraception use in the past month, consistency of use, experiences with violence, levels of depressive symptoms, self-esteem and sexual self-efficacy, substance use and health services utilization. Fifty percent of young sexually active women reported inconsistent or no contraception use in the past month. Inconsistent users were significantly more likely to report at least one prior episode of childhood sexual violence and were significantly less likely to have received a prescription for contraception from a health care provider. Inconsistent contraception users also reported significantly higher levels of depressive symptoms and significantly lower levels of self-esteem. The relation between childhood sexual violence and UP remained unchanged in the multivariate models adjusting for self-esteem or depressive symptoms. These findings highlight the long-term consequences of childhood sexual violence, independent of current depressive symptoms and low self-esteem, on consistent use of contraception.

## 1. Introduction

Unintended pregnancy (UP), defined as a mistimed or unwanted pregnancy, is a significant and prevalent public health problem [1,2]. Nationally, over one-half of all pregnancies are reported as unintended, and UP has been linked to numerous adverse outcomes including depression during pregnancy, delayed prenatal care, infant mortality, preterm birth, reduced breastfeeding and high levels of substance use [1,2,3,4,5]. Using consistent and effective contraception, including long acting reversible contraception (LARCs), is the primary method to prevent UP [1]. Although the Affordable Care Act of 2012 provides free contraception to women, removing financial barriers alone does not guarantee utilization of LARCs or consistent use of other effective methods of contraception [6,7].

High rates of unintended pregnancy occur among vulnerable women including young women, minority women, women living in poverty, women with multiple partners and women experiencing prior and current violence [2,8,9,10,11,12]. Violence is very prevalent among young urban women. According to the U.S. Department of Health and Human Services, one in five women has been a victim of childhood sexual abuse over the course of their lifetime, and 35.6% of women have experienced rape, physical violence and/or stalking by an intimate partner in their lifetime [13]. The majority of research to date has noted a role of experiences with childhood physical and sexual violence and high-risk sexual behaviors but these studies have not recognized the concurrent influence of depressive symptoms influencing high risk sexual behaviors [14,15,16,17]. Scant research has examined the role of childhood violence and inconsistent contraception use [18,19]. Allsworth *et al.*, described a cohort of young women enrolled in a project that provided contraception for free, and all women were given their contraception method of choice to examine the rate of contraception discontinuation [19]. This study found the highest rates of discontinuation of both LARC (17% *vs.* 14%, *p* = 0.04) and other non-LARC methods of contraception (56% *vs.* 47%, *p* < 0.001) among women reporting childhood physical or sexual violence; however, this study did not measure depressive symptoms or other psychological correlates of violence exposure.

Experiences of childhood sexual violence have been linked to higher depressive symptoms, lower sexual self-efficacy and reduced levels of self-esteem in adulthood [20,21]. Among adolescent females under Child Protective Services care in Quebec, childhood sexual abuse was negatively associated with adolescents’ sexual self-efficacy, perceived ability to communicate about their sexuality with their sexual partner, and contraceptive practices [20]. Depressive symptoms and reduced sexual self-efficacy following an experience of childhood sexual violence may impact on a young woman’s perceived competence in saying ‘no’ to sexual advances, her assertiveness in requiring contraceptive use during sex and her ability to discuss contraception use with a sexual partner or health care provider. Recognizing the intersection of childhood sexual violence and psychological factors, such as depressive symptoms, may be important when examining contraception use and risk of UP among young women.

The social ecological model provides a useful conceptual framework to identify and understand factors contributing to consistent and effective contraception use among young, sexually active women. The social ecological framework maintains that complex health-related behaviors are affected by multiple factors including those most proximal to the individual (e.g., psychological states such as depressive symptoms and self-esteem), social factors (e.g., interpersonal violence, childhood violence and community violence) to more distal factors (e.g., quality and delivery of health care systems) [22,23,24,25,26]. Current public health interventions designed to increase consistent contraception use and reduce UP have not incorporated this multiple factor approach but have targeted a change in discrete individual-level factors, such as decreasing the number of sexual partners [27]. Current interventions commonly use educational, counseling and motivational approaches that may not be appropriate for young sexually active women and do not recognize the role of violence exposures, depressive symptoms or self-esteem levels [27]. Limited efforts have focused on examining modifiable factors, such as depressive symptoms and self-esteem, within the context of violence exposures and consistent use of effective methods of contraception [28,29,30].

The present study investigated the role of childhood violence and current interpersonal violence, measured psychological correlates of violence exposure (high depressive symptoms and low level of self-esteem) and compared LARCs users, consistent users of other effective methods of contraception and inconsistent contraception users. We were particularly interested in assessing the role of childhood sexual violence, independent of current level of depressive symptoms and self-esteem, and hypothesized that recognizing current depressive symptoms or low self-esteem levels would reduce the role of childhood sexual violence on inconsistent contraception use. We were also interested in exploring whether chronic exposure to violence over a woman’s lifetime from childhood to adulthood influenced the consistency of contraceptive use.

## 2. Materials and Methods

### 2.1. Materials

#### 2.1.1. Enrollment and Eligibility

The Young Women’s Health Study (YWHS) is a cohort study of 315 urban women who sought care in Temple University family planning clinics from January 2013 through November 2013. Eligible women resided in North Philadelphia, were aged 18–30 years, reported sexual activity with a man in the past three months and were not currently pregnant or attending a *post-partum* visit. In addition, eligible women did not report a prior hysterectomy or tubal ligation. A total of 621 women were screened for study eligibility and 81% of the eligible women participated in the study (315 out of 390). The project enrolled women who reported sexual activity with a man in the last three months, since the goal was to prevent UP among current sexually active young women. The project focused on women living in North Philadelphia, a neighborhood located around Temple University hospital, which has particularly high rates of teen pregnancy and interpersonal and community violence [31]. Only the subgroup of women who reported at least one male sexual partner in the past three months and no pregnancy intention were included in the present analysis (*n* = 276). The Temple University Institutional Review Board (IRB) approved the study protocol and women were compensated thirty dollars for participating in this project.

#### 2.1.2. Data Collection

While in the waiting room, research coordinators approached each woman, described the purpose of the study and screened women for study eligibility. The waiting time in the clinic ranged from 30–60 min, and each questionnaire was completed prior to the clinic visit. An audio computer assisted survey instrument (ACASI) was used to collect information included in the questionnaire and to decrease bias when collecting sensitive information on prior and current violence. Each questionnaire was completed on a tablet, using a headset, thus participants were able to read and hear both the questions and the response options. The questionnaire collected information on demographic factors, current substance use, psychological factors such as depressive symptoms and self-esteem, childhood violence, current and past experiences with interpersonal violence, perceptions of community-level violence, health services utilization and contraception use in the past four weeks. The method of collecting the questionnaire on tablets, with required responses to proceed, resulted in no missing data elements.

#### 2.1.3. Measurement of Contraception Use

Reported contraception use in the past month was assessed at enrollment, and women were asked to report all methods of birth control in the past four weeks. Choices included birth control pills, withdraw/pull out, condoms, Depo-Provera, intrauterine device (IUD), patch, vaginal ring, Implanon, morning after pill, some other method, or I don’t use anything to prevent pregnancy. Women reporting the use of Depo-Provera, an IUD, patch, vaginal ring, or Implanon were classified as using LARCs (long acting reversible contraception), and these methods were considered consistent and effective. As described by others and used in the National Survey of Family Growth, women reporting other types of contraception were classified by the most effective contraceptive method used in the past month [29,32]. For example, if a woman reported taking the pill and using condoms she was classified as a pill user since the pill is more effective, and has a lower failure rate, compared to condoms. Two women reported the exclusive use of the morning after pill and they were included in the pill user group. To assess the consistency of contraception use in the past month, women were asked to think back over the past four weeks and report whether the birth control method was used every time, most of the time, about half of the time, some of the time, or none of the time. Pill and condom users reporting using this method of contraception during every sexual act with a man in the past month were classified as consistent users. Inconsistent users included women who were classified as pill users or condom users and did not report using this method of contraception every time, plus the group of women who reported using the withdrawal method or not using any form of contraception in the past month. Given the context of this assessment, to identify violence exposures and psychological factors related to consistent and effective contraception use, we described the demographic, violence and individual psychological constructs comparing women who reported LARC use (the group at lowest risk of UP), consistent use of non-LARC methods of contraception (the group at low risk of UP), and women reporting inconsistent use of non-LARC methods of contraception (the group at highest risk of UP).

#### 2.1.4. Individual Psychological Constructs

The 10-item Center for Epidemiologic Studies depression scale (CES-D) was used to measure depressive symptoms. This scale has been shown to be reliable among urban populations [33]. Questions include “I was bothered by things that usually do not bother me” and “I had trouble keeping my mind on what I was doing” (Cronbach’s alpha = 0.78). Higher mean scores on the CES-D indicated more depressive symptoms and the standard cut-off of 10 or higher was used to classify a woman as experiencing current depressive symptoms (y/n) [34]. An assessment of self-esteem using the reliable and validated Rosenberg self-esteem scale was administered, and higher scores indicated higher levels of self-esteem [35,36,37]. Questions include, “I feel that I have a number of good qualities’” and “All in all, I am inclined to feel that I am a failure” (Cronbach’s alpha = 0.78). Sexual self-efficacy was assessed using a modified version of the Sexual Self-Efficacy (SSE) Scale, a validated 20-item scale measuring perceptions of sexual health efficacy [38]. Questions include, “How certain do you feel in your ability to refuse a sexual advance by your partner” and “ How certain do you feel in your ability to have a sexual encounter without feeling obligated to have intercourse” with responses ranging from very uncertain, a little uncertain, sometimes certain, almost always certain, absolutely certain (Cronbach’s alpha = 0.95). High SSE scores indicate higher sexual self-efficacy. The five-item TWEAK (Tolerance, Worried, Eye-Opener, Amnesia, K/Cut) was used to detect alcoholism or heavy alcohol in-take [39]. The 12-item Social Provisions Scale (SPS) was used to measure social support received and provided by social networks, the modified SPS used in the study included the subscales of guidance, reliable alliances and social integration [40]. Questions include “There are people I can depend on to help me if I really need it” and “There is no one I can turn to for guidance in times of stress” (Cronbach’s alpha = 0.89). Higher scores of the SPS indicated higher perceived levels of overall social support.

#### 2.1.5. Childhood Violence Factors

Each woman was asked a series of questions concerning her experiences with childhood physical and sexual violence. Women were prompted to think about their entire childhood and remember all episodes of violence prior to 16 years of age. Women were asked two questions: “How often, before you were 16, were you slapped, pushed, hit, punched or beat up by someone you knew or by a stranger?” and “Before you were 16 years old, how often did anyone ever force you to have sex?” The response options for both questions consisted of never, once or twice, sometimes, often, and very often. Women reporting any amount of violence were classified as exposed to childhood physical or sexual violence and compared to women responding with not having experienced any violence. These questions have been used in other studies [41].

#### 2.1.6. Adult Violence Factors

Information concerning the amount of violence during adulthood, 16 years of age and older, as well as the relationship to the perpetrator was collected using standardized questions that have been used in other studies [41]. To assess adult physical violence, women were asked, “How often, in your lifetime, have you had an argument or fight with someone you knew or with a stranger?” and “During any argument or fight did you get slapped, pushed, hit, punched or beat up in anyway?” In this study, over 90% of women reported at least one episode of adult physical violence, much of the physical violence happened once or twice, and the proportion of adult physical violence did not differ between contraception users. Given the broadness of the adult physical violence question, the relation between adult physical violence and consistent contraception use was not assessed. To measure adult sexual violence, women were asked the question “Since you’ve been 16 years old, how often did anyone ever force you to have sex?” The response options included never, once or twice, sometimes, often, or very often, and women reporting any amount of sexual violence were classified as exposed and compared to women reporting none.

As described by Miller *et al.*, the degree of reproductive coercion from a sexual partner in the past year was collected and included measures of birth control sabotage and pregnancy coercion [42,43]. Birth control sabotage measured the intentional sabotage of contraception use by a sexual partner, and pregnancy coercion referred to a male sexual partner threatening to leave a woman if she did not become pregnant. The perception of current community level violence was collected using the City Stress Inventory (CSI), a validated 18-item scale to assess perceived neighborhood disorder and exposure to community-level violence and higher levels indicated a high perception of neighborhood violence [44].

#### 2.1.7. Health Services Utilization

Health services utilization in the past year was measured by four separate questions that asked women if they had received counseling or information about birth control in the past year, if they had received birth control or a prescription for birth control in the past year, if they had received emergency contraception or the morning-after pill in the past year, and if they had any health problems for which they would have liked to have seen a doctor or other medical person, but did not.

### 2.2. Statistical Methods

Descriptive statistics were generated for the total sample and by type of contraception user (LARCs, consistent non-LARC user and inconsistent user). Student’s two-sample *t*-tests/one-way ANOVAs or nonparametric Wilcoxon tests were used to compare groups by continuous risk factors. Contingency tables and measures of association were used to compare categorical variables with Fishers exact tests or chi-square tests employed to assess statistical significance. Due to sample size constraints, multiple logistic regression models compared inconsistent contraception users to consistent contraception users. For the multiple logistic regression assessments, women reporting LARC use and women reporting consistent use of other non-LARC forms of contraception were combined to create the consistent contraception use group. A two-tailed p-value of 0.05 was considered statistically significant and variables significant at a *p*-value of 0.05 or less were included in the multiple regression model. Pearson’s correlation coefficients were used to assess the correlation between important psychological factors, and significantly correlated factors were not included in the same regression model. SPSS version 22 was used for these analyses.

## 3. Results

As shown in Table 1, participants were mostly single, young, minority women whose highest level of education was high school graduation or less. Thirty-one percent of women reported LARC use, 18% reported consistent use of other effective methods of contraception (*i.e.*, pills and condoms), and fifty percent of women reported inconsistent or no contraception use in the past month. We found no difference in the mean age, racial composition, or other behavioral factors between the groups. Women reporting inconsistent contraception use were more likely to be classified as a problem drinker (22.2%) but this finding was not significantly different across the groups (*p* = 0.09). We did find that women reporting inconsistent contraception use were significantly less likely to have received a prescription for contraception from a health care provider compared to women reporting LARC or consistent contraception use (33.8% *vs.* 87.2% or 68.6%, respectively; *p*-value < 0.001) although access to a health care provider in the past year was similar between the groups (Table 1).

Women reporting inconsistent contraception use reported significantly higher levels of prior childhood sexual violence compared to women reporting consistent contraceptive use and women reporting LARC use (23.7% *vs.* 17.6% and 7.0%, respectively, *p*-value = 0.05). The level of childhood physical violence was similar across the groups. Current interpersonal violence was high in all three groups and the proportion of women reporting at least one episode of prior adult sexual violence was dramatically lower among women reporting LARC use, although none of these differences were statistically significant (Table 1). A higher proportion of women reporting inconsistent contraception use reported being forced to have sex without a condom by their sexual partner in the past year compared to women reporting consistent contraception use and LARC use (15.1% *vs.* 7.8% and 7.0%, respectively *p* = 0.04) (Table 1). We did not find a difference in the perception of community level violence between the three groups.

In this cohort, the level of self-esteem was related to consistent contraception use with significantly lower mean self-esteem scores among women reporting inconsistent contraception use compared to women reporting consistent contraception use or LARC use (22.5 ± 5.7 *vs.* 23.4 ± 5.0 and 24.3 ± 5.3 respectively, *p*-value = 0.023) (Table 1). In addition, women reporting inconsistent contraception use were more likely to report high depressive symptoms compared to women reporting consistent or LARC use (48.9% v. 35.3% and 37.2% respectively, *p*-value = 0.037). Sexual self-efficacy and social support were not related to contraception use. As shown in Table 2, we found the level of self-esteem was significantly correlated with the report of sexual self-efficacy (r = 0.143, *p* = 0.01) and depressive symptoms (r = −0.603, *p*-value < 0.001), thus self-esteem and depressive symptoms were not included in the same multiple regression models.

**Table 1:** Characteristics of contraception use among young, urban, sexually active women.

	Total Sample (*n* = 276)	Long Acting Reversible Contraception Users (*n* = 86)	Consistent Contraception Users (*n* = 51)	Inconsistent Contraception Users (*n* = 139)
Demographic Factors				
Age ^1,^* (in years)	22.6	22.1 ± 3.1	23.3 ± 2.9	22.6 ± 3.2
Race ^2^				
Black	79.7%	77.9%	80.4%	80.6%
White	3.6%	1.2%	5.9%	4.3%
Other	16.7%	20.9%	13.7%	15.1%
Hispanic origin ^2^	16.3%	20.9%	15.7%	13.7%
Education level ^2^				
Less than High School	22.5%	24.4%	15.7%	23.7%
High School Graduate	50.4%	47.7%	52.9%	51.1%
Some College/College Graduate	27.2%	27.9%	31.4%	25.2%
Relationship Status ^2^				
Single, dating more than 1 person	27.9%	23.3%	41.2%	25.9%
Single, in a serious relationship	59.1%	65.1%	49.0%	59.0%
Other	13.0%	11.6%	9.8%	15.1%
Received contraception prescription, past yearr ^2,^*^,††^	56.9%	87.2%	68.6%	33.8%
Number of lifetime sexual partners ^2^				
1–4	39.5%	45.3%	37.3%	36.7%
5–8	29.7%	26.7%	41.2%	27.3%
9–19	18.8%	19.8%	9.8%	21.6%
20+	12.0%	8.1%	11.8%	14.4%
Problem drinker, past year ^2^	17.5%	15.5%	8.6%	22.2%
Marijuana use, past year ^2^	40.6%	43.0%	29.4%	43.2%
Current smoking	27.2%	25.6%	21.6%	30.2%
Ever Homeless ^2^	19,9%	14.0%	19.6%	23.7%
Access to health care provider, past yr ^2^	80.1%	83.7%	80.4%	77.7%
Violence Factors				
*Overall Reproductive Sabotage*	28.6%	25.6%	27.5%	30.9%
Having sex without a condom ^2,††^	11.3%	7.0%	7.8%	15.1%
Taking off a condom ^2^	23.6%	22.1%	15.7%	27.3%
Breaking a condom ^2^	4.7%	2.3%	5.9%	5.8%
Childhood physical violence, ever ^2^	47.8%	47.7%	49.0%	47.5%
Child sexual violence, ever ^2,^*^,††^	17.4%	7.0%	17.6%	23.7%
Adult sexual violence, eve ^2^	17.8%	10.5%	21.6%	20.9%
Perceptions of Community Violence ^1^	38.3	37.4 ± 11.7	38.8 ± 11.9	38.6 ± 11.2
Current Interpersonal Violence	67.0%	64.0%	66.7%	69.1%
Individual Constructs				
Depressive symptoms ^1,^*	9.35 ± 5.55	9.09 ± 5.2	8.30 ± 5.3	9.91 ± 5.8
Depressive symptoms ^2,^*^,††^	42.8%	37.2%	35.3%	48.9%
Self-esteem ^1,†^	23.2	24.3 ± 5.3	23.4 ± 5.0	22.5 ± 5.7
Sexual self-efficacy ^1^	60.2	61.5 ± 15.7	58.3 ± 19.0	60.1 ± 15.5
Social Provisions Scale ^1^	29.9	29.8 ± 2.6	29.7 ± 1.9	29.9 ± 2.6

Problem drinker assessed using the T_1_weak; 1: Continuous variable: Mean (std); 2: Categorical variable: Percentage; †: Significant Wilcoxon rank sum; *: Overall χ2 significant; ††: χ2 significant compared inconsistent users to other.

**Table 2:** Correlation Coefficients.

		Social Provisions Scale	Sexual Self-Efficacy	Self-Esteem	Depressive Symptoms
Social Provisions Scale	Pearson Correlation	1	−0.080	−0.085	0.064
	Sig. (2-tailed)		0.158	0.131	0.259
Sexual self-efficacy	Pearson Correlation	−0.080	1	0.143 *	−0.072
	Sig. (2-tailed)	0.158		0.011	0.201
Self-esteem	Pearson Correlation	−0.085	0.143 *	1	−0.603 **
	Sig. (2-tailed)	0.131	0.011		0.000
Depressive symptoms	Pearson Correlation	0.064	−0.072	−0.603 **	1
	Sig. (2-tailed)	0.259	0.201	0.000	

Multivariate analysis continued to find a significant relation between prior experiences with childhood sexual violence and inconsistent contraception use adjusting for level of self-esteem and receiving a contraception prescription (aOR = 2.21, 95% CI: 1.04–4.71) (Table 3a). The childhood sexual violence finding also remained significant in the separate model adjusting for current depressive symptoms and receiving a contraception prescription (aOR = 2.19, 95% CI: 1.03–4.67) (Table 3b). In both multiple regression models, receiving a prescription for contraception decreased the odds of inconsistent contraception use by 87% (Table 3a and 3b). Neither self-esteem nor depressive symptoms remained significant in the multivariate models. We were interested in examining the role of chronic violence exposure and contraception use and found that women reporting childhood sexual violence were significantly more likely to report adult violence with 85% of women reporting childhood sexual violence also reporting physical violence in the past year. A composite factor was created to compare women reporting both a history of childhood sexual violence and adult physical violence in the past year to women reporting no violence over their lifetime. Higher but non-significant increased odds of inconsistent contraception use was found in the model including the chronic violence composite measure, level of self-esteem and receiving a prescription. For women reporting both childhood sexual violence and adult physical violence in the past year, a two-fold increase odds of inconsistent contraception use was noted (aOR = 2.21, 95% CI: 0.89–5.54).

**Table 3:** Multivariate models. (**a)** Multivariate model predicting inconsistent *vs.* consistent contraception users among young, urban women; (**b)** multivariate model predicting inconsistent *vs.* consistent contraception users among young, urban Women.

**(a)** Multivariate model including self-esteem			
	**aOR**	**95% CI**	***p*-Value**
Received contraception prescription in past year	0.13	0.08–0.23	<0.001
Level of Self-Esteem	0.97	0.93–1.03	0.32
Child Sexual Violence	2.21	1.04–4.71	0.039
**(b)** Multivariate model including depressive symptoms			
	**aOR**	**95% CI**	***p*-Value**
Received contraception prescription in past year	0.13	0.07–0.22	<0.001
Level of depressive symptoms	1.03	0.97–1.08	0.32
Child Sexual Violence	2.19	1.03–4.67	0.04

## 4. Discussion

These findings expand the examination of the role of violence and consistent contraception use by recognizing the psychological correlates of violence, high depressive symptoms and low self-esteem, and assessing contraception use among a sample of young urban women seeking care in a family planning clinic. We hypothesized that current depressive symptoms and low self-esteem would reduce the relation between childhood sexual violence and inconsistent contraception use, but found that the experience of childhood sexual violence remained after accounting for these important psychological correlates. This is one of the first studies to examine the role of exposure to childhood sexual violence and consistent contraception use among a clinic-based population. In addition, this manuscript recognizes the influence of psychological correlates of violence (*i.e.*, high depressive symptoms and low self-esteem) on the relation between violence and contraception use. Others have linked childhood exposures to adverse adult outcomes [45,46,47,48,49]. In particular, the adverse childhood exposures (ACEs) study reported adverse childhood exposures, including childhood sexual violence, related to a higher risk of sexually transmitted diseases, reports of unintended pregnancy, high-risk sexual behaviors and teen pregnancy.

These results suggest a strong relation between history of childhood sexual violence and inconsistent contraception use independent of level of self-esteem or depressive symptoms. Given these findings, it is clear that experiences with childhood sexual violence have long-term and lasting consequences above the increase in depressive symptoms and lower self-esteem. In this study, we found that women with a history of childhood sexual violence reported that they were less likely to feel certain about their ability to refuse a sexual advance by their partner, reported a lower ability to have a sexual encounter without feeling obligated to have intercourse, and had a lower ability to choose when and with whom to have sex. Perhaps exposures to childhood sexual violence impact a young woman’s ability to comfortably discuss sexual desires and to demand contraception use with a sexual partner. Building strong negotiation skills among women who have experienced childhood sexual violence may promote consistent contraception use among this high-risk group of young women.

We also found that women reporting inconsistent contraception use were less likely to receive a prescription for contraception from a health provider but reported similar access to health care. Callegari *et al.*, examined data from the National Survey of Family Growth and found that compared to obese women who reported effective contraception use, obese women who reported less effective methods of contraception were significantly less likely to report discussing contraception with a health care provider in the past year [50]. In fact, conversations regarding contraception with a health care provider have been related to an increase in the use of more effective types of contraception including hormonal contraception, LARCs and the vaginal ring [51,52,53]. Future initiatives to reduce UP should focus on improving the dialogue between young sexually active women and their health care provider about the various types of effective contraception methods. This simple but important intervention may increase LARC use among women who may be uncomfortable discussion contraception use with a sexual partner or promote consistent effective use of other effective methods of contraception [54]. These results also highlight the importance of health care providers routinely screening women for prior childhood violence when discussing contraception options.

We initially found the level of self-esteem and depressive symptoms, two modifiable factors, related to inconsistent contraception use but this effect was reduced in the multivariate models. High depressive symptoms have been related to both inconsistent contraceptive use and lower odds of choosing an effective method of contraception in some but not all studies [55,56,57]. Hall *et al.*, found that the odds of weekly consistent contraceptive use were reduced by 47% in women with high depressive symptoms (OR = 0.53, 95% CI: 0.31–0.91) [55]. However, Callegari *et al.*, found that female veterans with mental illness, including depression, posttraumatic stress disorder, anxiety, bipolar disorder, schizophrenia and adjustment disorder, were more likely to report the use of effective prescription methods, and Farr *et al.*, found that women with frequent mental distress had higher odds of more permanent and effective types of contraception [56,58]. More recently, Steinberg *et al.*, reported that women experiencing more psychological distress before an abortion selected more effective contraceptive methods after their abortion, however the consistent use of contraception was not assessed in this study [59]. It is important to recognize that these prior studies linking depressive symptoms to contraception use did not measure childhood or current violence exposures. Clearly, further research is needed to determine the complicated and ongoing role of depressive symptoms and level of self-esteem, within the context of violence exposure and consistent contraception use.

Limitations exist in this study and need to be recognized. First, this study lacked a male perspective, which is a valuable piece in understanding sexual partner relationship barriers to consistent contraception use. The ability to discuss contraception use, negotiate consistent contraception use and agree on the amount and type of sexual activity are skills both young women and men need to develop to promote consistent contraception use and the reduction of UP. The importance of strong contraceptive communication skills between couples may be an important factor contributing to consistent contraception use. Unfortunately, this factor was not measured in the present study. Second, we did not find perception of community level violence, recent reproductive coercion or interpersonal violence related to UP as reported by others [60,61,62,63,64]. O’Hara *et al.*, reported that women who reported ever having experienced severe physical violence from an intimate partner were significantly less likely to use contraception, and others have reported a role of experiences with reproductive coercion and reports of inconsistent contraception use and risk of UP [60,63,64]. It is important to note that the questions used to assess physical violence were very inclusive and may have included less severe forms of physical violence, which may have resulted in the null findings for childhood physical and adult physical violence. By design, all women included in the study were from North Philadelphia, a community with high rates of violence and UP although the prevalence of childhood physical and sexual violence found in this study (40% and 17%, respectively) parallel the high rates of childhood violence documented in other studies of urban women [45,49]. Third, the sample size of women included in this assessment was limited and specific to young urban women, reducing the generalizability of these findings and the ability to confer a causal inference.

## 5. Future Directions

These findings highlight the long-term consequences of childhood sexual violence on consistent contraception use and the importance of health care providers routinely screening for childhood sexual violence when discussing contraception options. Recognizing the role of experiences with childhood sexual violence, independent of depressive symptoms and level of self-esteem, may inform trauma-sensitive interventions which are targeted and more acceptable to young, urban, sexually active women to promote consistent and effective contraception use.
